# Longitudinal Assessments of Neurocognitive Performance and Brain Structure Associated With Initiation of Tobacco Use in Children, 2016 to 2021

**DOI:** 10.1001/jamanetworkopen.2022.25991

**Published:** 2022-08-10

**Authors:** Hongying Daisy Dai, Gaelle E. Doucet, Yingying Wang, Troy Puga, Kaeli Samson, Peng Xiao, Ali S. Khan

**Affiliations:** 1 College of Public Health, University of Nebraska Medical Center, Omaha; 2 Brain Architecture, Imaging and Cognition Laboratory, Boys Town National Research Hospital, Omaha, Nebraska; 3 Neuroimaging for Language, Literacy & Learning Laboratory, University of Nebraska at Lincoln; 4 Bioinformatics and Systems Biology Core, University of Nebraska Medical Center, Omaha

## Abstract

**Importance:**

The landscape of tobacco use is changing. However, information about the association between early-age tobacco use and cognitive performances is limited, especially for emerging tobacco products such as electronic cigarettes (e-cigarettes).

**Objective:**

To assess the association between early-age initiation of tobacco use and cognitive performances measured by the National Institutes of Health (NIH) Toolbox Cognitive Battery and to examine whether initiation is associated with differences in brain morphometry.

**Design, Setting, and Participants:**

This observational cohort study examined the longitudinal associations of initiation of tobacco use with neurocognition using multivariate linear mixed models. Children aged 9 to 10 years from 21 US sites were enrolled in wave 1 (October 1, 2016, to October 31, 2018 [n = 11 729]) and the 2-year follow-up (August 1, 2018, to January 31, 2021 [n = 10 081]) of the Adolescent Brain Cognitive Development (ABCD) Study.

**Exposures:**

Ever use (vs none) of any tobacco products at wave 1, including e-cigarettes, cigarettes, cigars, smokeless tobacco, hookah, pipes, and nicotine replacement.

**Main Outcomes and Measures:**

Neurocognition measured by the NIH Toolbox Cognition Battery and morphometric measures of brain structure and region of interest analysis for the cortex from structural magnetic resonance imaging.

**Results:**

Among 11 729 participants at wave 1 (mean [SE] age, 9.9 [0.6] years; 47.9% girls and 52.1% boys; 20.3% Hispanic; 14.9% non-Hispanic Black; and 52.1% non-Hispanic White), 116 children reported ever use of tobacco products. Controlling for confounders, tobacco ever users vs nonusers exhibited lower scores in the Picture Vocabulary Tests at wave 1 (*b* [SE] = −2.9 [0.6]; *P* < .001) and 2-year follow-up (*b* [SE] = −3.0 [0.7]; *P* < .001). The crystalized cognition composite score was lower among tobacco ever users than nonusers both at wave 1 (*b* [SE] = −2.4 [0.5]; *P* < .001) and 2-year follow-up (*b* [SE] = −2.7 [0.8]; *P* = .005). In structural magnetic resonance imaging, the whole-brain measures in cortical area and volume were significantly lower among tobacco users than nonusers, including cortical area (*b* [SE] = −5014.8 [1739.8] mm^2^; *P* = .004) at wave 1 and cortical volume at wave 1 (*b* [SE] = −174 621.0 [5857.7] mm^3^; *P* = .003) and follow-up (*b* [SE] = −21 790.8 [7043.9] mm^3^; *P* = .002). Further region of interest analysis revealed smaller cortical area and volume in multiple regions across frontal, parietal, and temporal lobes at both waves.

**Conclusions and Relevance:**

In this cohort study, initiating tobacco use in late childhood was associated with inferior cognitive performance and reduced brain structure with sustained effects at 2-year follow-up. These findings suggest that youths vulnerable to e-cigarettes and tobacco products should be treated as a priority population in tobacco prevention.

## Key Points

**Question:**

How is initiation of tobacco use, including electronic cigarettes, cigarettes, and other tobacco products, at an early age associated with children’s neurocognitive functions?

**Findings:**

In this national cohort study of 17 073 children with neuroimaging outcomes, a significant association was found of early-age initiation of tobacco use with lower crystalized cognition composite score and impaired brain development in total cortical area and volume. Region of interest analysis also revealed smaller cortical area and volume across frontal, parietal, and temporal lobes.

**Meaning:**

These findings suggest that initiation of tobacco use is associated with inferior neurocognitive functions; evidence-based intervention strategies and tobacco control policies should prevent tobacco initiation at a young age.

## Teaser

This national cohort study assesses the association between early-age initiation of tobacco use and cognitive performances measured by the National Institutes of Health Toolbox Cognitive Battery and examines whether tobacco use is associated with differences in brain morphometry among children aged 9 to 10 years.

## Introduction

The landscape of tobacco use has substantially changed in the last decade, with more adolescents using electronic cigarettes (e-cigarettes) and other noncigarette tobacco products. Electronic cigarettes are battery-powered devices that people can use to inhale an aerosol via a heating element.^1^ Use of e-cigarettes has surpassed use of cigarettes and become the most prevalent tobacco product used by US youths since 2014.^2,3^ The latest generation of e-cigarette products has a sleek design and comes in various flavors appealing to youths (eg, candy, mint, fruit).^4^ In 2020, 19.6% of high school students and 4.7% of middle school students reported using e-cigarettes in the past 30 days.^5^ Other alternative tobacco products, including cigars, hookah, and smokeless tobacco products, have also gained popularity among US adolescents. In 2020, cigars (3.5%) ranked the second most used tobacco product after e-cigarettes among US adolescents. The prevalence of current smokeless tobacco (2.3%) and hookah (2.1%) use was not far different from that of cigarette smoking (3.3%).^6^ Importantly, alternative tobacco products are often marketed as odorless and less harmful than combustible cigarettes.^7^ These marketing strategies could lower harm perception and increase curiosity among adolescents, making inroads to tobacco use among substance-naive children.^8^

Nicotine concentration levels doubled from 2013 to 2018 with rapidly evolving e-cigarette products.^9^ Biospecimen analysis in 2017 to 2018 showed adolescents who used vaping pod systems (such as Juul) were exposed to a high concentration of nicotine (244.8 ng/mL),^10^ with the mean concentration level higher than a 2013-2014 study of adolescent smokers (155.2 ng/mL).^11^ Nicotine and other chemicals inherent to e-liquids and aerosols may cause neurotoxic effects on the developing brain.^12^

Childhood to early adolescence represents a critical period of intersection when youths undergo multiple stages of brain development concomitant with an increase in risk-taking behaviors.^13^ Youths initiate tobacco use as early as 7 years of age,^14,15^ and early initiation of use of tobacco products (<13 vs ≥13 years of age) could increase the risk of nicotine dependence and sustained tobacco use.^16^ Youth with lower socioeconomic status are of particular concern for initiation of tobacco use at an early age because the health of these children is already disproportionately affected by other social determinants of health (eg, economic impoverishment or neighborhood disadvantage).^17^ Studies have shown the association between low socioeconomic status and early age at initiation of tobacco use^18^ and smoking during adolescence^19^ increases the risk for the development of psychiatric disorders and cognitive impairment. However, most of the current epidemiological studies have focused on youths 12 years or older,^1,15^ and longitudinal behavioral-neurocognitive research on early-age initiation of tobacco use is scarce.

Youth nicotine use can lead to addiction and harm the developing brain, impairing learning, memory, and attention and causing impulsivity, irritability, anxiety, and poor decision making.^15,19^ Children are at increased risk for these adverse effects because they have an increased reward response in the striatal regions of the brain to stimuli.^20^ The existing work on nicotine addiction and neurocognitive assessment has primarily been focused on use of combustible cigarettes, adult tobacco users, or animal models in laboratory settings.^1,21,22^ Limited research has shown that neurotoxic chemicals, particles, and free radicals have been found in e-cigarette aerosols and liquids in addition to nicotine.^23^ Nicotine concentrations, doses, and formulation often have a wide variation by type of tobacco product, brand, and device, which could cause differential neurocognitive effects for users at different ages. Animal models have demonstrated these differential neurocognitive effects for users at different ages in a dose-dependent manner.^24^ However, information about the outcomes associated with early-age tobacco use and cognitive performances is limited, especially for emerging tobacco products such as e-cigarettes. It is critical to measure neurocognitive functions by objective measures and gauge the health effects of initiation of tobacco use at the population level, by considering demographic characteristics and socioecological factors.

This study analyzed the longitudinal data from the Adolescent Brain and Cognitive Development (ABCD) Study^25^ to (1) assess the association between early-age initiation of tobacco use and cognitive performances measured by the National Institutes of Health (NIH) Toolbox Cognitive Battery; and (2) examine whether ever tobacco use is associated with differences in brain morphometry. We focused on ever use of tobacco as a measure for initiation of tobacco use in the early stage of life because the first use of tobacco is symbolically significant with an enduring life-long impact.^26,27,28^ We hypothesized that early-age initiation of tobacco use would be associated with lower cognitive performance cross-sectionally at wave 1 and prospectively at 2-year follow-up (wave 2).

## Methods

### Data and Participants

This study analyzed the ABCD data, version 4.0, released by the National Data Archive. The ABCD Study is a large cohort that enrolled children aged 9 and 10 years at wave 1 across 21 US research sites between October 1, 2016, and October 31, 2018.^29^ Wave 1 participants were recruited through a probability sample of schools selected for sex at birth, race and ethnicity, socioeconomic status, and urbanicity.^30^ Race and ethnicity were derived from self-reported responses to the ABCD survey. Participants and at least 1 parent or guardian completed a comprehensive battery including clinical interviews, surveys, neurocognitive tests, and neuroimaging.^30,31,32^ All parents or guardians provided written informed consent, and children gave written assent. Participants are asked for in-person assessment sessions once a year and brain imaging biannually.^33^ The follow-up cognitive performance and brain imaging were measured between August 1, 2018, and January 31, 2021. The ABCD Study procedure was approved by the centralized institutional review board of the University of California, San Diego, and by the institutional review boards at each local institution. This report followed the Strengthening the Reporting of Observational Studies in Epidemiology (STROBE) guideline for cohort studies.

### Measures

#### Tobacco Ever Use (Wave 1)

In the timeline flow-back survey, participants were first asked whether they had heard of tobacco products, such as cigarettes, smokeless tobacco, cigars, hookah, or e-cigarettes. Those who reported yes were further asked whether they have ever tried any tobacco products in their life by separate questions for each type of product (ie, e-cigarette, cigarette, cigar, smokeless tobacco, hookah, pipe, and nicotine replacement). Those who reported yes were classified as ever users of tobacco.

The NIH Toolbox Cognition Battery at both waves includes the Dimensional Change Card Sort, Flanker Inhibitory Control and Attention, List Sorting Working Memory, Oral Reading Recognition, Pattern Comparison Processing Speed, Picture Sequence Memory, and Picture Vocabulary tests.^34,35^ Composite cognitive scores include total, crystallized, and fluid scores.^36^

#### Magnetic Resonance Neuroimaging Measures at Both Waves

Magnetic resonance imaging (MRI) methods and assessments were optimized and harmonized across ABCD Study sites for 3T scanners.^25^ Participants completed MRI scanning of 3-dimensional T1-weighted images. Cortical surface reconstruction and subcortical segmentation were processed through FreeSurfer, version 5.3.0,^37^ using the standardized ABCD pipeline.^38,39^ For this study, we specifically focused on the 34 cortical measures of surface area and volumes from the DKT (Desikan-Killany-Tourville) atlas.^38^ We specifically chose cortical volume and surface area metrics because they have partially distinct evolutionary,^40^ genetic,^41^ developmental,^42^ and environmental correlates.^43^

Quality control of MRI image processing includes a manual review of images for artifacts (eg, wraparound, missing brain image due to improper section prescription, signal dropout due to magnetic susceptibility artifacts, and motion). Participants with poor quality of neuroimaging in the ABCD MRI data were removed from the analysis. Details of MRI image acquisition and quality control are provided in the appendix of the previous study by Vidal-Ribas et al.^44^ Wave 1 sociodemographic characteristics included age, sex at birth, race and ethnicity, parent’s highest educational level, family income, and experience of family difficulty in the past 12 months.

#### Wave 1 Substance Use and Other Influencing Factors

Children self-reported ever use of alcohol, cannabis products, and other illicit drugs (eg, cocaine, methamphetamine, ecstasy [MDMA], ketamine, γ-hydroxybutyrate, heroin, psilocybin, salvia, other hallucinogens, anabolic corticosteroids, inhalants, prescription stimulants, sedatives, opioid pain relievers, and over-the-counter cough or cold medicine).^45^ Children self-rated a puberty development scale,^46^ which consists of 5 questions regarding changes in height, body hair, skin, voice, and facial hair (boys) or breast development and menarche (girls) (Cronbach α = 0.67). A higher puberty development scale mean score (range, 1-4) indicates a more mature level of perceived physical development. The child-reported parent monitoring scale consisted of 5 items (eg, “How often do your parents know where you are?”) (Cronbach α = 0.50).^47^ Higher scores indicate stronger parental monitoring. The School Risk and Protective Factors School Environment subscale consisted of 6 items (eg, “I get along with my teachers.”) (Cronbach α = 0.62).^48^ Higher scores indicate a favorable school environment.

### Inclusion and Exclusion Criteria

**Figure 1.**
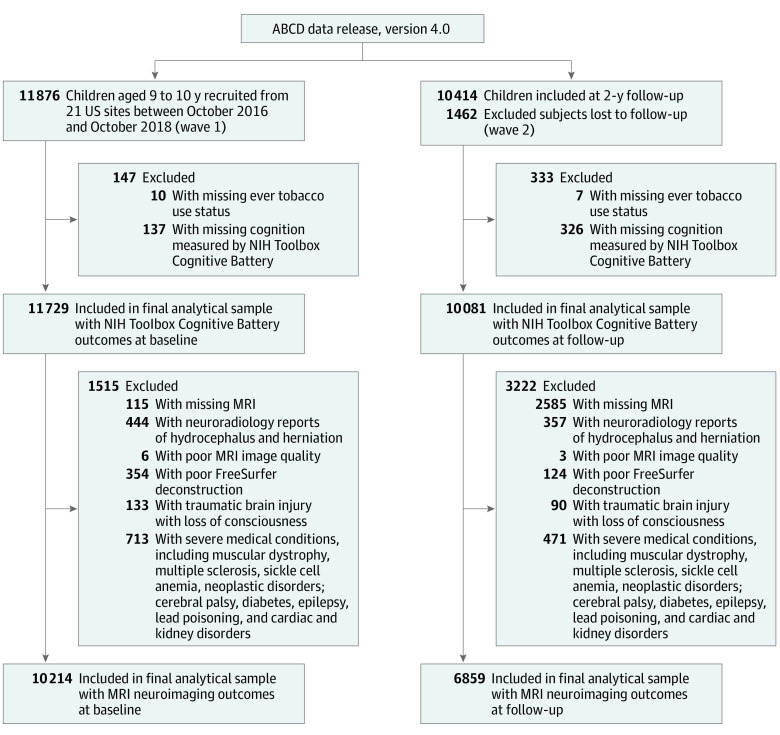
Flowchart of Analytical Sample Inclusion and Exclusion From the Adolescent Brain Cognitive Development (ABCD) Study The selection procedure was based on the protocols in previous studies and the ABCD Study magnetic resonance imaging quality control guideline.^44,49^ NIH indicates National Institutes of Health.

As illustrated in Figure 1, the ABCD Study included 11 876 participants enrolled at wave 1 (2016-2018) and 10 414 participants at 2-year follow-up (2018-2021) with a retention rate of 87.7%. After excluding participants with missing variables, poor MRI images, and medical reasons, the final analytical sample for MRI neuroimaging outcomes included 10 214 participants for wave 1 and 6859 participants for wave 2. The selection criteria was based on the ABCD Study MRI quality control guidelines and protocols in our previous studies.^44,49^ Details are found in eMethods in the Supplement.

### Statistical Analysis

Participant characteristics were reported by tobacco ever use status at wave 1 and the 2-year follow-up. Following the statistical guide for the population-based analysis of ABCD Study, weighted analysis was conducted to account for the clustering of participants across 21 study sites, sample selection biases, and nonresponsiveness in the observational study design.^50^ The weight variable was generated using a propensity model of age, sex, and race and ethnicity with missing data imputation to ensure that weighted ABCD data maintain the sample demographic profiles in accordance with the American Community Survey third and fourth grade enrollment statistics at each site.^30,50^ Study sites were included as clusters in survey analytical procedures. Separate multivariable linear mixed models were built to examine the associations of tobacco ever use with the NIH Toolbox Cognition Battery measures and with brain morphometric measures, adjusted by age, sex assigned at birth, race and ethnicity, ever use of other substances, youth pubertal stage, parent monitoring, and school environment. A sensitivity analysis was performed to further account for intracranial volume. The structural MRI (sMRI) neuroimaging analyses included investigating associations between tobacco ever use and whole-brain and regional measures of 34 cortical structures (surface area and volume, analyzed separately) across frontal, parietal, temporal, and occipital lobes. In sensitivity analysis, we performed stratified assessments of sMRI global morphometric measures within the left and right hemispheres. Handedness (left vs right) and MRI device manufacturer were also included in the multivariable regression models of morphometric measures.^51^ Using generalized least square estimation, we estimated adjusted regression coefficient *b* and SE for associations of wave 1 tobacco ever use and the outcomes at both wave 1 and the 2-year follow-up. The variance-covariance matrix for the regression coefficients was estimated by the Taylor series method. Statistical analyses were conducted in SAS, version 9.4 (SAS Institute, Inc). Two-sided *P* < .05 indicated statistical significance with a Benjamin-Hochberg multiple test correction to control the study-wise false discovery rate.^52^

## Results

The overall sample included 11 729 participants at wave 1 (mean [SE] age, 9.9 [0.6] years) (eTable 1 in the Supplement). The wave 1 participants were sociodemographically diverse, including 47.9% girls and 52.1% boys. In regard to race and ethnicity, 2.1% were Asian; 20.3%, Hispanic; 14.9%, non-Hispanic Black; and 52.1%, non-Hispanic White. For socioeconomic characteristics, 27.3% had parents who were college graduates; 31.8%, parents with postgraduate degrees; and 13.7%, parents with annual income less than $25 000. In wave 1, 22.7% of participants had self-reported use of other substances.

Among 116 children who reported ever use of tobacco products, 80 reported use of e-cigarettes or cigarettes. Other tobacco products included cigars (reported by 10 participants), smokeless tobacco (reported by 12 participants), hookah (reported by 7 participants), pipes (reported by 5 participants), and nicotine replacement (reported by 8 participants). These subsamples were not mutually exclusive owing to dual use of tobacco products. Weighted sample characteristics, such as sex (eg, 51.1% [95% CI, 50.2%-52.0%] vs 58.8% [95% CI, 51.4%-66.2%] boys; *P* = .04) and ever use of other substances (eg, 21.7% [95% CI, 18.5%-24.9%] vs 59.9% [95% CI, 51.9%-67.9%]; *P* < .001), were significantly different between wave 1 tobacco ever users and nonusers (eTable 1 in the Supplement).

Table 1 presents the comparison of cognitive performances between tobacco ever users and nonusers. At wave 1, tobacco ever users (vs nonusers) exhibited lower scores on the Picture Vocabulary Test (mean [SE] *b* = −2.9 [0.6]; *P* < .001). Compared with nonusers, tobacco ever users had lower crystalized cognition composite scores (*b* [SE] = −2.4 [0.5]; *P* < .001) and total cognition composition scores (*b* [SE] = −2.9 [0.5]; *P* = .01). At the 2-year follow-up, wave 1 tobacco ever users continued to exhibit lower scores for Oral Reading Recognition (*b* [SE] = −2.1 [0.5]; *P* < .001), Picture Sequence Memory (*b* [SE] = −5.4 [1.8]; *P* = .007), and Picture Vocabulary Test (*b* [SE] = −3.0 [0.7]; *P* < .001) and lower crystalized cognition composite scores (*b* [SE] = −2.7 [0.8]; *P* = .005) than nonusers.

**Table 1.** Comparison of Cognitive Performances Between Tobacco Ever Users and Nonusers^a^

NIH Toolbox Cognition Battery subscale	Study period							
	Wave 1				Wave 2			
	Nonuse, weighted mean (SE) (n = 11 613)	Tobacco ever use, weighted mean (SE) (n = 116)	Adjusted *b* (SE)^b^	Adjusted *P* value^c^	Nonuse, weighted mean (SE) (n = 10 117)	Tobacco ever use, weighted mean (SE) (n = 86)	Adjusted *b* (SE)^b^	Adjusted *P* value^c^
Dimensional Charge Card Sort	92.3 (0.3)	91.5 (1)	−0.4 (1.5)	.77	NA	NA	NA	NA
Flanker Inhibitory Control and Attention	93.8 (0.3)	94 (0.7)	0.2 (1.0)	.83	99.9 (0.3)	100 (1.4)	0.2 (1.9)	.91
List Sorting Working Memory	96.1 (0.5)	93.9 (1)	−2.1 (1.4)	.16	NA	NA	NA	NA
Oral Reading Recognition	90.6 (0.2)	88.7 (0.6)	−1.5 (0.6)	.03	94.7 (0.3)	93.1 (0.5)	−2.1 (0.5)	<.001^d^
Pattern Comparison Process Speed	88 (0.5)	84.8 (1.2)	−3.5 (1.6)	.04	103.3 (0.5)	101 (1.7)	−2.0 (2.3)	.40
Picture Sequence Memory	102.6 (0.4)	100.1 (0.8)	−2.7 (1.2)	.04	108.5 (0.4)	104.7 (2)	−5.4 (1.8)	.007^d^
Picture Vocabulary Tests	84 (0.5)	82 (0.8)	−2.9 (0.6)	<.001^d^	88.5 (0.5)	87 (1.1)	−3.0 (0.7)	<.001^d^
Crystalized cognition composite score	86 (0.3)	83.8 (0.5)	−2.4 (0.5)	<.001^d^	90.5 (0.4)	89.3 (0.8)	−2.7 (0.8)	.005^d^
Fluid cognition composite score	91.2 (0.5)	88.7 (0.7)	−2.4 (1.3)	.08	NA	NA	NA	NA
Total cognition composite score	85.8 (0.5)	83 (0.6)	−2.9 (0.5)	.01^d^	NA	NA	NA	NA

Abbreviations: NA, not available; NIH, National Institutes of Health.

^a^ Multivariate regression analyses were performed where the dependent variables were cognitive performance scores. Sampling weights and site clustering were incorporated in the survey regression analytical procedures for statistical inference at the population level. The independent variable was early age at initiation of tobacco use (ever use) measured at wave 1.

^b^ The analysis was adjusted by covariates, including age, sex, race and ethnicity, pubertal stage, substance ever use, parental monitoring, school environment, and study site. A sensitivity analysis was performed to further account for intracranial volume (eTable 4 in the Supplement). Regression coefficients measured the association of early-age initiation of tobacco use as changes in cognitive performance scores between tobacco ever users vs controls (no use).

^c^ False discovery rate (FDR) correction was performed to prevent inflation of type I errors.

^d^ Indicates FDR of less than 0.05.

**Figure 2.**
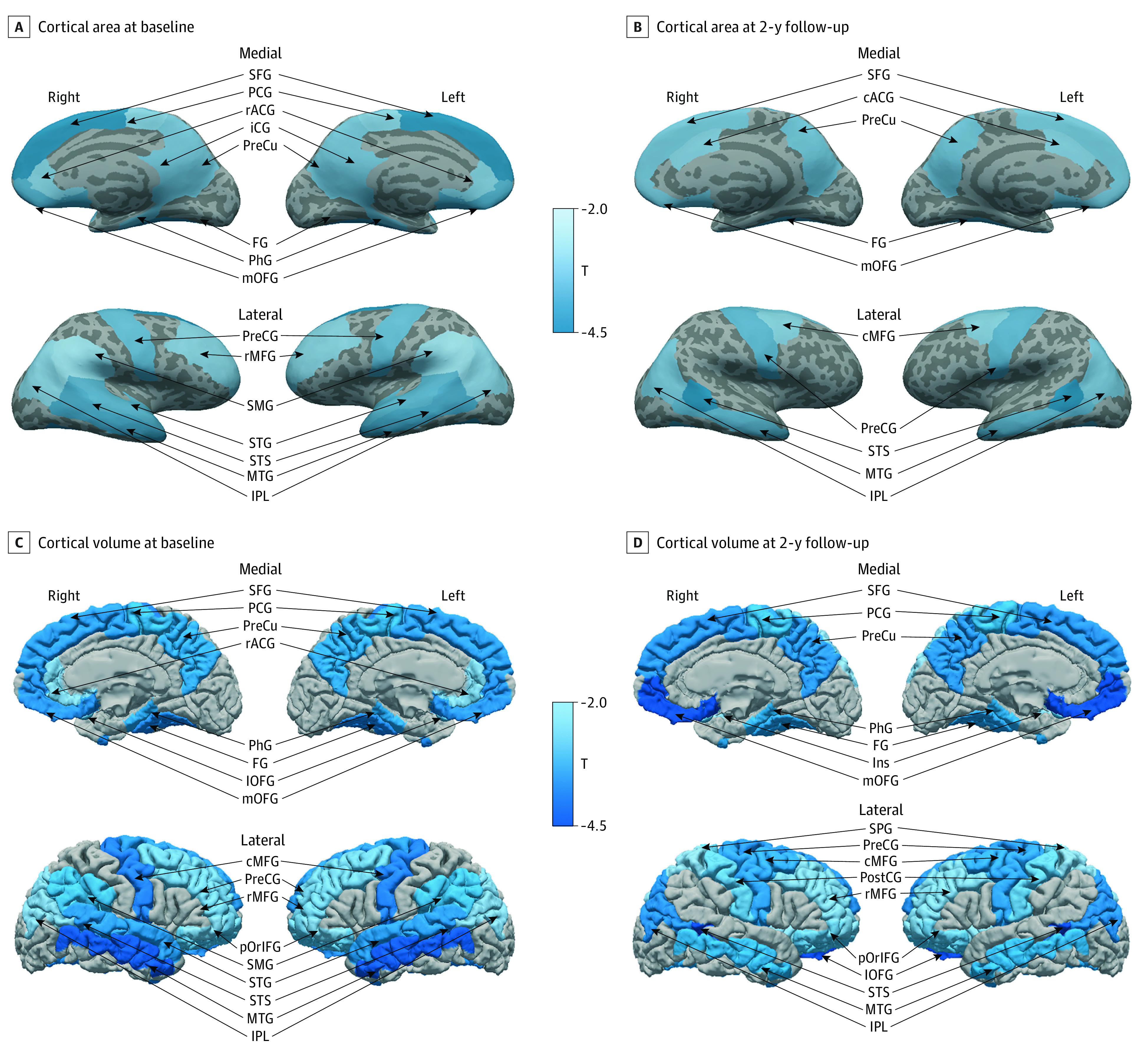
Differential Brain Structures Associated With Initiation of Tobacco Use in Childhood Cortical surface measurements include 15 clusters at baseline and 10 clusters at 2-year follow-up. Cortical volume measurements include 17 clusters at baseline and 2-year follow-up. Brain structures significantly associated with early initiation of tobacco use (false discovery rate <0.05) are labeled by T values of cortical area and volume between ever tobacco users and nonusers, adjusted by age, sex, race and ethnicity, pubertal stage, substance ever use, parental monitoring, school environment, handedness, imaging device manufacturer, and study site. cACG indicates caudal anterior cingulate gyrus; cMFG, caudal middle frontal gyrus; FG, fusiform gyrus; iCG, isthmus cingulate; Ins, insula; lOFG, lateral orbitofrontal gyrus; mOFG, medial orbitofrontal gyrus; MTG, middle temporal gyrus; IPL, inferior parietal lobule; PCG, paracentral gyrus; PhG, parahippocampal gyrus; pOrIFG, pars orbitalis; PostCG, postcentral gyrus; PreCG, precentral gyrus; PreCu, precuneus; rACG, rostral anterior cingulate gyrus; rMFG, rostral middle frontal gyrus; SFG, superior frontal gyrus; SMG, supramarginal gyrus; STG, superior temporal gyrus; and STS, banks of superior temporal sulcus.

In sMRI analyses (Table 2 and Figure 2), multiple whole-brain measures were significantly lower among tobacco ever users than nonusers, including total cortical surface area at wave 1 (*b* [SE] = −5014.8 [1739.8] mm^2^; *P* = .004) and cortical volume at wave 1 (*b* [SE] = −174 621.0 [5857.7] mm^3^; *P* = .003) and at follow-up (*b* [SE] = −21 790.8 [7043.9] mm^3^; *P* = .002) and total intracranial volume (*b* [SE] = −38 442.8 [12 057.7] mm^3^; *P* = .009) at wave 1. Within each hemisphere, tobacco ever users also exhibited lower global measures at both left and right hemispheres (ie, cortical surface area and cortical volume) (eTable 2 in the Supplement).

**Table 2.** Comparison of sMRI Morphometric Measures Between Tobacco Ever Users and Nonusers^a^

Whole brain measures	Study period							
	Wave 1				Wave 2			
	Nonuse, weighted mean (SE) (n = 10 117)	Tobacco ever use, weighted mean (SE) (n = 97)	Adjusted *b* (SE)^b^	*P* value^c^	Nonuse, weighted mean (SE) (n = 6806)	Tobacco ever use, weighted mean (SE) (n = 53)	Adjusted *b* (SE)^b^	*P* value^c^
Total cortical surface area, mm^2^	188 723.0 (942.5)	185 088.0 (2570.2)	−5014.8 (1739.8)	.004^d^	189 511.0 (940.5)	186 833.0 (3217.0)	−5377.5 (2376.9)	.02
Mean cortical thickness, mm	2.7 (0)	2.7 (0)	0 (0)	.48	2.7 (0)	2.7 (0)	0 (0)	.16
Total cortical volume, mm^3^	594 869.0 (2423.9)	582 577.0 (7694.5)	−174 621.0 (5857.7)	.003^d^	586 304.0 (2669.8)	573 498.0 (11 038.0)	−21 790.8 (7043.9)	.002^d^
Subcortical gray matter volume, mm^3^	59 777.0 (162.9)	59 114.0 (600.6)	−786.8 (537.3)	.14	60 533.0 (207.1)	60 441.0 (776.9)	−589.9 (695.8)	.40
Cerebral white matter volume, mm^3^	418 363.0 (1790.6)	412 615.0 (6132.0)	−8457.3 (4696.8)	.07	432 787.0 (1797.2)	430 719.0 (9166.7)	−8115.7 (6661.9)	.22
Intracranial volume, mm^3^	1 485 762.0 (12 664.0)	1 458 959.0 (12 647.0)	−38 442.8 (12 057.7)	.009^d^	1 515 063.0 (13 920.0)	1 490 582.0 (15 605.0)	−42 938.1 (22 037.7)	.05

Abbreviation: sMRI, structural magnetic resonance imaging.

^a^ Multivariate regression analyses were performed where the dependent variables were whole-brain measures. Sampling weights were incorporated in the survey regression analytical procedures for statistical inference at the population level. The independent variable was early age initiation of tobacco use (ever use) measured at wave 1.

^b^ Adjusted by covariates, including age, sex, race and ethnicity, pubertal stage, substance ever use, parental monitoring, school environment, handedness, imaging device manufacturer, and study site. A sensitivity analysis was performed to further account for intracranial volume (eTable 4 in the Supplement). Regression coefficients measured the association of early-age tobacco initiation as changes in sMRI measures between tobacco ever users vs control (no use).

^c^ False discovery rate (FDR) testing correction was performed to prevent inflation of type I errors.

^d^ Indicates FDR less than 0.05.

Further region-of-interest analyses of cortical surface area and volume are presented in Table 3 and eTable 3 in the Supplement. In the 2-year follow-up, tobacco ever users had lower surface area in superior frontal (*b* = −927.1 [SE, 325.1]; *P* = .004), caudal middle (*b* = −337.1 [SE 130.1]; *P* = .01), medial orbitofrontal (*b* = −164.7 [SE, 58.8]; *P* = .005), precentral (*b* = −439.7 [SE, 133.8]; *P* = .001), caudal anterior (*b* = −112.5 [SE, 42.9]; *P* = .009), inferior parietal (*b* = −606.5 [SE, 216.0]; *P* = .005), precuneus (*b* = −411.3 [SE, 140.0]; *P* = .003), middle temporal (*b* = −443.1 [141.0]; *P* = .002), banks of superior temporal sulcus (*b* = −145.7 [SE, 36.0]; *P* < .001), and fusiform gyri (*b* = −334.3 [SE, 98.8]; *P* < .001) than nonusers. In 2-year follow-up, tobacco ever users had lower cortical volume in frontal (eg, superior frontal, medial orbitofrontal, paracentral), parietal (eg, inferior parietal), and temporal (eg, middle temporal, banks of superior temporal sulcus, fusiform gyri) lobes than nonusers.

**Table 3.** Region of Interest Analysis of Cortical Surface Between Tobacco Ever Users and Nonusers^a^

Cortical surface	Study period									
	Wave 1					Wave 2				
	Nonuse, weighted mean (SE), mm^2^ (n = 10 117)	Tobacco ever use, weighted mean (SE), mm^2^ (n = 97)	Adjusted *b*^b^	Adjusted *P* value	FDR^c^	Nonuse, weighted mean (SE), mm^2^ (n = 6806)	Tobacco ever use, weighted mean (SE), mm^2^ (n = 53)	Adjusted *b*^b^	Adjusted *P* value	FDR^c^
Frontal										
Superior frontal	15 934.0 (87.9)	15 468.0 (209.5)	−744.4 (255.4)	.004	0.02	16 096.0 (86.9)	15 598.0 (306.7)	−927.1 (325.1)	.004	0.02
Rostral middle	13 522.0 (85.9)	13 261.0 (214.2)	−497.9 (197.3)	.01	0.03	13 594.0 (81.7)	13 447.0 (295.2)	−371.4 (258.7)	.15	0.24
Caudal middle	4912.8 (43.5)	4807.9 (101.6)	−226.9 (104.0)	.03	0.06	4969.6 (45)	4793.3 (156.3)	−337.1 (130.1)	.01	0.03
Pars opercularis	3320.4 (19.9)	3266.2 (70.6)	−87.4 (77.8)	.26	0.32	3339.3 (19.0)	3323.2 (78.6)	−89.5 (94.4)	.34	0.42
Pars triangularis	3305.8 (16.4)	3281.3 (66.0)	−47.8 (57.5)	.41	0.44	3315.2 (17.2)	3325.6 (73.7)	−46.9 (64.0)	.46	0.52
Pars orbitalis	1747.4 (7.7)	1731.4 (26.2)	−43.5 (26.4)	.10	0.15	1760.3 (9.2)	1751.3 (37.1)	−55.8 (36.5)	.13	0.21
Lateral orbitofrontal	5727.9 (29.7)	5632.2 (68.6)	−146.5 (69.9	0.04	0.06	5789.1 (35.0)	5680.2 (83.3)	−169.3 (92.6)	.07	0.15
Medial orbitofrontal	4093.6 (22.1)	4004.2 (48.1)	−157.1 (46.5)	<.001	0.01	4131.9 (25.4)	4037.3 (60.9)	−164.7 (58.8)	.01	0.02
Precentral	10 207.0 (72.0)	10 054.0 (140.0)	−364.3 (114.0)	.001	0.01	10 309.0 (78.3)	10 149.0 (224.7)	−439.7 (133.8)	.001	0.01
Paracentral	3042.7 (10.3)	2965.9 (44.3)	−93.1 (36.3)	.01	0.03	3049.7 (11.8)	2980.6 (52.0)	−111.9 (51.8)	.03	0.09
Frontal pole	674.4 (2.8)	673.8 (9.0)	−13.1 (8.7)	.13	0.18	676.2 (3.1)	675.0 (12.3)	−19.0 (12.3)	.12	0.21
Rostral anterior	1570.2 (7.3)	1541.6 (23.8)	−75.9 (29.4)	.01	0.03	1596.1 (8.0)	1584.7 (33.6)	−65.7 (40.5)	.10	0.21
Caudal anterior	1485.0 (6.9)	1450.9 (41.5)	−72.8 (32.2)	.02	0.05	1514.8 (7.3)	1461.2 (52.4)	−112.5 (42.9)	.009	0.03
Parietal										
Superior parietal	11 896.0 (91.9)	11 788.0 (241.4)	−239.3 (163.7)	.14	0.19	11 839.0 (96.1)	11 821.0 (228.1)	−349.7 (198.6)	.08	0.17
Inferior parietal	11 224.0 (69.2)	10 885.0 (200.6)	−417.6 (167.4)	.01	0.03	11 167.0 (68.4)	10 771.0 (242.7)	−606.5 (216.0)	.005	0.02
Supramarginal	8629.3 (71.1)	8345.8 (151.6)	−372.1 (161.7)	.02	0.05	8613.1 (66.3)	8631.4 (175.4)	−239.1 (216.5)	.27	0.37
Postcentral	8770.0 (71.3)	8706.6 (212.1)	−147.4 (117.9)	.21	0.27	8773.5 (75.8)	8698.5 (243.5)	−292.7 (130.7)	.03	0.08
Precuneus	8616.4 (31.2)	8412.9 (125.2)	−356.0 (126.1)	.005	0.02	8559.0 (36.3)	8401.4 (104.2)	−411.3 (140.0)	.003	0.02
Posterior cingulate	2682.6 (9.3)	2624.3 (41.2)	−86.9 (41.2)	.035	0.06	2687.5 (10.6)	2659.1 (53.3)	−84.5 (62.5)	.18	0.27
Isthmus cingulate	2157.0 (7.1)	2100.7 (35.5)	−93.1 (32.8)	.005	0.02	2156.6 (8.5)	2123.0 (40.4)	−62.8 (39.3)	.11	0.21
Temporal										
Superior temporal	8443.4 (53.3)	8142.7 (118.0)	−389.4 (116.5)	<.001	0.01	8455.4 (46.8)	8345.4 (192.6)	−202.9 (160.8)	.21	0.29
Inferior temporal	7336.6 (45.8)	7250.2 (127.5)	−200.1 (105.8)	.06	0.09	7424.2 (46.1)	7307.6 (184.9)	−278.7 (138.5)	.04	0.12
Middle temporal	7635.3 (58.5)	7339.2 (165.3)	−394.5 (109.4)	<.001	0.01	7688.8 (57.1)	7407.6 (211.0)	−443.1 (141.0)	.002	0.01
Banks of superior temporal sulcus	2169.8 (15.8)	2064.8 (42.0)	−113.8 (32.2)	<.001	0.01	2165.4 (14.7)	2065.4 (53.5)	−145.7 (36.0)	<.001	0.002
Fusiform	6625.1 (20.1)	6466.4 (82.2)	−279.6 (72.9)	<.001	0.004	6678.9 (21.0)	6506.8 (129.3)	−334.3 (98.8)	<.001	0.01
Transverse temporal	841.7 (3.0)	819.5 (10.4)	−26.4 (13.4)	.05	0.08	840.6 (2.9)	841.1 (16.2)	−8.5 (19.5)	.66	0.70
Entorhinal	837.0 (4.6)	844.9 (14.8)	6.6 (20.9)	.75	0.75	848.9 (4.6)	845.5 (20.5)	−24.1 (23.2)	.30	0.39
Temporal pole	994.0 (3.5)	992.6 (11.5)	−18.9 (11.2)	.09	0.14	1002.9 (4.4)	1020.2 (18.4)	−6.1 (15.6)	.69	0.71
Parahippocampal	1314.7 (4.2)	1271.5 (15)	−58.9 (20.8)	.01	0.02	1334.2 (4.4)	1298.1 (15.7)	−55.5 (29.6)	.06	0.15
Occipital										
Lateral occipital	11 026.0 (62.3)	11 009.0 (176.9)	−139.6 (147.0)	.34	0.39	11 030.0 (64.3)	11 077.0 (228.8)	−208.6 (209.9)	.32	0.40
Lingual	6701.1 (25.4)	6604.6 (86.9)	−150.4 (97.1)	.12	0.17	6735.1 (33.0)	6759.2 (93.5)	−104.8 (136.2)	.44	0.52
Cuneus	3374.7 (13.8)	3408.8 (46.7)	21.3 (52.1)	.68	0.72	3383.7 (15.1)	3434.5 (53.1)	34.1 (76.7)	.66	0.70
Pericalcarine	3184.6 (13.8)	3208.2 (55.8)	−24.5 (65.5)	.71	0.73	3206.1 (16.9)	3259.6 (57.7)	13.8 (102.0)	.89	0.89
Insula	4721.2 (16.6)	4663.6 (45.5)	−66.4 (60.4)	.27	0.32	4780.3 (18.4)	4753.2 (49)	−87.7 (68.6)	.20	0.29

Abbreviation: FDR, false discovery rate.

^a^ Multivariate regression analyses were performed where the dependent variables were cortical surface areas in 34 regions of interest listed in the first column. The independent variable was early age at initiation of tobacco use (ever use) measured at wave 1.

^b^ Adjusted by covariates, including age, sex, race and ethnicity, pubertal stage, substance ever use, parental monitoring, school environment, handedness, imaging device manufacturer, and study site. Sampling weights were incorporated in the survey regression analytical procedures for statistical inference at the population level. Regression coefficients measured the outcomes associated with early-age initiation of tobacco use as changes in structural magnetic resonance imaging measures between tobacco ever users vs control (nonuse).

^c^ FDR correction was performed across 34 regions to prevent inflation of type I errors.

## Discussion

This cohort study sheds light on the association of health effects with early initiation of tobacco use, including e-cigarettes, cigarettes, and other tobacco products. These results are consistent with results from animal models that indicate that a short exposure period or low plasma nicotine concentration could lead to lasting cellular and neuritic damages.^53^

Our results showed that tobacco ever users continued to have significantly lower scores in higher-order cognitive functions, particularly in oral reading recognition, auditory comprehension, and crystallized intelligence, compared with nonusers. These cognitive functions relate to verbal and reading capacity and are more dependent on past learning experiences.^36^ The Picture Vocabulary Test measures receptive vocabulary by asking participants to select the right image for words with varying difficulty levels; the Oral Reading Recognition subdomain is a cognitive test of language; and blunted language development has demonstrated a decrease in IQ scores and difficulty with later language development and comprehension ability.^54^ Crystalized abilities are typically based on previous vocabulary and reading skills, which may be necessary for success in future schoolwork.^36^

Using brain cortical morphometric measures, our results robustly showed that tobacco ever users had smaller brain cortical volumes, both at the whole brain and regional levels. In particular, associative brain areas such as the superior frontal gyri, lateral temporal cortices, and inferior parietal lobes were consistently found to be smaller at wave 1 and 2-year follow-up. These regions have been linked to language functions,^55^ which may explain our findings with crystallized cognition. In particular, the lateral temporal cortex houses the arcuate fasciculus, an important connection of neurons between the primary center of language comprehension (Wernicke area) and the primary speech production center (Broca area).^56,57^ The arcuate fasciculus is essential for the cognitive function of speech, comprehension, and word retrieval.^56^ Decreased cortical area in the lateral temporal cortex in this study may correlate to decreased function of the arcuate fasciculus and therefore decreased language abilities in tobacco ever users. Future long-term studies need to determine whether these decreased language skills can be overcome or are irreversible consequences of early age tobacco use.

As children develop, the total cortical area continues to increase in size into early adolescence.^58^ Because the associative cortex (ie, frontal, parietal, and temporal cortices) is essential for higher order cognition and continues development into early adolescence, the decreased cortical surface area and cortical volume exhibited by tobacco ever users during this critical development period may be of concern.

Our regional analyses also revealed smaller cortical areas and volumes in widespread areas across the frontal, parietal, and temporal lobes. Our findings are in line with those of a recent study that described thinner frontal cortex in adolescents who smoke, relative to nonsmokers.^59^ The current findings further highlight that experimental use of tobacco products during childhood may have severe long-term structural consequences on brain morphometry. The prefrontal cortex is still largely under development during adolescence. We found early initiation of tobacco use was associated with lower area and volume in the prefrontal cortex, which could suppress executive functions and attention performance and increase the risk of developing psychiatric disorders and cognitive impairment in later life.^19^

The findings from the present study could be interpreted in 2 intertwined directions. The tobacco industry heavily promotes marketing campaigns, social media, and various emerging tobacco products to entice a new generation of users, increasing the susceptibility of children with existing impaired cognition to initiate tobacco use. In contrast, the adolescent brain is sensitive to nicotine neurotoxicity, causing impaired cognition. A growing body of literature has identified tobacco-related health disparities and emerging racial disparities in trends of e-cigarette use among youths.^60,61^ Socioeconomic status and other influencing factors (eg, parental monitoring and school environment) could interact with pediatric nicotine exposure on brain maturation. Future studies should examine whether socioeconomic status might moderate the association between tobacco use by youths and neurocognitive performances.

### Limitations

This study has limitations. First, the ABCD Study participants are not fully nationally representative, with a paucity of rural families. However, it is a national and diverse study across 21 US sites, with the study sample designed to be epidemiologically informed and to minimize selection bias.^62^ Sampling weight was incorporated into the analysis to reduce the selection bias. Second, causal inference cannot be established based on this observational study. However, we adjusted multiple covariates (eg, sociodemographic characteristics and ever substance use) and further excluded potential confounders in the MRI analyses (eg, those with traumatic brain injury and severe medical conditions). Furthermore, this study examined associations between tobacco ever use and neurocognition in 2 waves. Future studies should test whether tobacco use is associated with changes in cognitive performances and brain functions. Third, tobacco use status was self-reported, subject to social desirability biases, especially for younger respondents.^63^ However, the test and retest reliability of self-reported behaviors associated with tobacco use among adolescents is high.^63^ Last, we focused on specific cortical morphometric measures (surface area and volumes). Future studies should test other sMRI metrics and other neuroimaging measures (eg, task-based functional, resting state, and diffusion MRI) to determine their unique patterns of covariation with tobacco use.

## Conclusions

Results of this cohort study suggest that initiation of tobacco use in late childhood at 9 to 10 years of age is associated with inferior cognitive performance and brain development with sustained effects at the 2-year follow-up. Electronic cigarettes and smokeless tobacco products should not be treated as harm reduction alternatives for youth. Comprehensive intervention strategies and tobacco control policies are needed to prevent tobacco initiation.

## Notes

Supplement. **eMethods.** Participant Inclusion and Exclusion Criteria **eTable 1.** Participant Characteristics **eTable 2.** Comparison of sMRI Morphometric Measures Within Cerebral Hemisphere Between Tobacco Ever Users and Nonusers **eTable 3.** Region of Interest Analysis of Cortical Volume Between Tobacco Ever Users and Nonusers **eTable 4.** Sensitivity Analysis of Cognitive Performances and sMRI Morphometric Measures With Adjustment of Intracranial Volume
eReferences

## References

[1] Centers for Disease Control and Prevention. 2016 Surgeon General’s report: e-cigarette use among youth and young adults. Accessed July 8, 2022. https://www.cdc.gov/tobacco/sgr/e-cigarettes/index.htm#report 30869850

[2] Gentzke AS, Creamer M, Cullen KA, . Vital signs: tobacco product use among middle and high school students—United States, 2011-2018. MMWR Morb Mortal Wkly Rep. 2019;68(6):157-164. doi:10.15585/mmwr.mm6806e1 30763302 PMC6375658

[3] Miech R, Johnston L, O’Malley PM, Bachman JG, Patrick ME. Trends in adolescent vaping, 2017-2019. N Engl J Med. 2019;381(15):1490-1491. doi:10.1056/NEJMc1910739 31532955 PMC7310772

[4] Barrington-Trimis JL, Leventhal AM. Adolescents’ use of “Pod Mod” e-cigarettes—urgent concerns. N Engl J Med. 2018;379(12):1099-1102. doi:10.1056/NEJMp1805758 30134127 PMC7489756

[5] Wang TW, Neff LJ, Park-Lee E, Ren C, Cullen KA, King BA. E-cigarette use among middle and high school students—United States, 2020. MMWR Morb Mortal Wkly Rep. 2020;69(37):1310-1312. doi:10.15585/mmwr.mm6937e1 32941408 PMC7498174

[6] Gentzke AS, Wang TW, Jamal A, . Tobacco product use among middle and high school students—United States, 2020. MMWR Morb Mortal Wkly Rep. 2020;69(50):1881-1888. doi:10.15585/mmwr.mm6950a1 33332300 PMC7745956

[7] Gorukanti A, Delucchi K, Ling P, Fisher-Travis R, Halpern-Felsher B. Adolescents’ attitudes towards e-cigarette ingredients, safety, addictive properties, social norms, and regulation. Prev Med. 2017;94:65-71. doi:10.1016/j.ypmed.2016.10.019 27773711 PMC5373091

[8] Vasiljevic M, St John Wallis A, Codling S, Couturier D-L, Sutton S, Marteau TM. E-cigarette adverts and children’s perceptions of tobacco smoking harms: an experimental study and meta-analysis. BMJ Open. 2018;8(7):e020247. doi:10.1136/bmjopen-2017-020247 30012646 PMC6082488

[9] Romberg AR, Miller Lo EJ, Cuccia AF, . Patterns of nicotine concentrations in electronic cigarettes sold in the United States, 2013-2018. Drug Alcohol Depend. 2019;203:1-7. doi:10.1016/j.drugalcdep.2019.05.029 31386973 PMC6765364

[10] Goniewicz ML, Boykan R, Messina CR, Eliscu A, Tolentino J. High exposure to nicotine among adolescents who use Juul and other vape pod systems (‘pods’). Tob Control. 2019;28(6):676-677. doi:10.1136/tobaccocontrol-2018-054565 30194085 PMC6453732

[11] Benowitz NL, Nardone N, Jain S, . Comparison of urine 4-(methylnitrosamino)-1-(3) pyridyl-1-butanol and cotinine for assessment of active and passive smoke exposure in urban adolescents. Cancer Epidemiol Biomarkers Prev. 2018;27(3):254-261. doi:10.1158/1055-9965.EPI-17-0671 29475963 PMC5835192

[12] Ruszkiewicz JA, Zhang Z, Gonçalves FM, Tizabi Y, Zelikoff JT, Aschner M. Neurotoxicity of e-cigarettes. Food Chem Toxicol. 2020;138:111245. doi:10.1016/j.fct.2020.111245 32145355 PMC7089837

[13] Nock NL, Minnes S, Alberts JL. Neurobiology of substance use in adolescents and potential therapeutic effects of exercise for prevention and treatment of substance use disorders. Birth Defects Res. 2017;109(20):1711-1729. doi:10.1002/bdr2.1182 29251846 PMC5751741

[14] Chen X, Yu B, Wang Y. Initiation of electronic cigarette use by age among youth in the US. Am J Prev Med. 2017;53(3):396-399. doi:10.1016/j.amepre.2017.02.011 28372920

[15] National Center for Chronic Disease Prevention and Health Promotion (US) Office on Smoking and Health. Preventing tobacco use among youth and young adults: a report of the Surgeon General. 2012. Accessed July 8, 2022. https://www.ncbi.nlm.nih.gov/books/NBK99237/22876391

[16] Campaign for Tobacco-Free Kids. The path to tobacco addiction starts at very young ages. December 15, 2021. Accessed July 2, 2022. https://www.tobaccofreekids.org/assets/factsheets/0127.pdf

[17] Viner RM, Ozer EM, Denny S, . Adolescence and the social determinants of health. Lancet. 2012;379(9826):1641-1652. doi:10.1016/S0140-6736(12)60149-4 22538179

[18] Wellman RJ, Sylvestre MP, O’Loughlin EK, . Socioeconomic status is associated with the prevalence and co-occurrence of risk factors for cigarette smoking initiation during adolescence. Int J Public Health. 2018;63(1):125-136. doi:10.1007/s00038-017-1051-9 29116338

[19] Goriounova NA, Mansvelder HD. Short- and long-term consequences of nicotine exposure during adolescence for prefrontal cortex neuronal network function. Cold Spring Harb Perspect Med. 2012;2(12):a012120. doi:10.1101/cshperspect.a012120 22983224 PMC3543069

[20] Padmanabhan A, Geier CF, Ordaz SJ, Teslovich T, Luna B. Developmental changes in brain function underlying the influence of reward processing on inhibitory control. Dev Cogn Neurosci. 2011;1(4):517-529. doi:10.1016/j.dcn.2011.06.004 21966352 PMC3181104

[21] National Center for Chronic Disease Prevention and Health Promotion (US) Office on Smoking and Health. The health consequences of smoking—50 years of progress: a report of the Surgeon General. 2014. Accessed July 8, 2022. https://www.ncbi.nlm.nih.gov/books/NBK179276/

[22] Chamberlain SR, Odlaug BL, Schreiber LR, Grant JE. Association between tobacco smoking and cognitive functioning in young adults. Am J Addict. 2012;21(suppl 1):S14-S19. doi:10.1111/j.1521-0391.2012.00290.x 23786505

[23] Goniewicz ML, Kuma T, Gawron M, Knysak J, Kosmider L. Nicotine levels in electronic cigarettes. Nicotine Tob Res. 2013;15(1):158-166. doi:10.1093/ntr/nts103 22529223

[24] Torres OV, Natividad LA, Tejeda HA, Van Weelden SA, O’Dell LE. Female rats display dose-dependent differences to the rewarding and aversive effects of nicotine in an age-, hormone-, and sex-dependent manner. Psychopharmacology (Berl). 2009;206(2):303-312. doi:10.1007/s00213-009-1607-3 19629450 PMC2746680

[25] Casey BJ, Cannonier T, Conley MI, ; ABCD Imaging Acquisition Workgroup. The Adolescent Brain Cognitive Development (ABCD) study: Imaging acquisition across 21 sites. Dev Cogn Neurosci. 2018;32:43-54. doi:10.1016/j.dcn.2018.03.001 29567376 PMC5999559

[26] Breslau N, Peterson EL. Smoking cessation in young adults: age at initiation of cigarette smoking and other suspected influences. Am J Public Health. 1996;86(2):214-220. doi:10.2105/AJPH.86.2.214 8633738 PMC1380330

[27] DeBry SC, Tiffany ST. Tobacco-induced neurotoxicity of adolescent cognitive development (TINACD): a proposed model for the development of impulsivity in nicotine dependence. Nicotine Tob Res. 2008;10(1):11-25. doi:10.1080/14622200701767811 18188741

[28] DeLorme DE, Kreshel PJ, Reid LN. Lighting up: young adults’ autobiographical accounts of their first smoking experiences. Youth Soc. 2003;34(4):468-496. doi:10.1177/0044118X03034004004

[29] Volkow ND, Koob GF, Croyle RT, . The conception of the ABCD study: From substance use to a broad NIH collaboration. Dev Cogn Neurosci. 2018;32:4-7. doi:10.1016/j.dcn.2017.10.002 29051027 PMC5893417

[30] Garavan H, Bartsch H, Conway K, . Recruiting the ABCD sample: Design considerations and procedures. Dev Cogn Neurosci. 2018;32:16-22. doi:10.1016/j.dcn.2018.04.004 29703560 PMC6314286

[31] Barch DM, Albaugh MD, Avenevoli S, . Demographic, physical and mental health assessments in the adolescent brain and cognitive development study: Rationale and description. Dev Cogn Neurosci. 2018;32:55-66. doi:10.1016/j.dcn.2017.10.010 29113758 PMC5934320

[32] Feldstein Ewing SW, Chang L, Cottler LB, Tapert SF, Dowling GJ, Brown SA. Approaching retention within the ABCD Study. Dev Cogn Neurosci. 2018;32:130-137. doi:10.1016/j.dcn.2017.11.004 29150307 PMC6333413

[33] Karcher NR, Barch DM. The ABCD study: understanding the development of risk for mental and physical health outcomes. Neuropsychopharmacology. 2021;46(1):131-142. doi:10.1038/s41386-020-0736-6 32541809 PMC7304245

[34] Gershon RC, Slotkin J, Manly JJ, . IV. NIH Toolbox Cognition Battery (CB): measuring language (vocabulary comprehension and reading decoding). Monogr Soc Res Child Dev. 2013;78(4):49-69. doi:10.1111/mono.12034 23952202 PMC7659464

[35] Zelazo PD, Anderson JE, Richler J, Wallner-Allen K, Beaumont JL, Weintraub S. II. NIH Toolbox Cognition Battery (CB): measuring executive function and attention. Monogr Soc Res Child Dev. 2013;78(4):16-33. doi:10.1111/mono.12032 23952200

[36] Akshoomoff N, Beaumont JL, Bauer PJ, . VIII. NIH Toolbox Cognition Battery (CB): composite scores of crystallized, fluid, and overall cognition. Monogr Soc Res Child Dev. 2013;78(4):119-132. doi:10.1111/mono.12038 23952206 PMC4103789

[37] Laboratory for Computational Neuroimaging. FreeSurfer software suite. Accessed July 8, 2022. https://surfer.nmr.mgh.harvard.edu/

[38] Desikan RS, Ségonne F, Fischl B, . An automated labeling system for subdividing the human cerebral cortex on MRI scans into gyral based regions of interest. Neuroimage. 2006;31(3):968-980. doi:10.1016/j.neuroimage.2006.01.021 16530430

[39] Hagler DJ Jr, Hatton S, Cornejo MD, . Image processing and analysis methods for the Adolescent Brain Cognitive Development Study. Neuroimage. 2019;202:116091. doi:10.1016/j.neuroimage.2019.116091 31415884 PMC6981278

[40] Geschwind DH, Rakic P. Cortical evolution: judge the brain by its cover. Neuron. 2013;80(3):633-647. doi:10.1016/j.neuron.2013.10.045 24183016 PMC3922239

[41] Chen C-H, Fiecas M, Gutiérrez ED, . Genetic topography of brain morphology. Proc Natl Acad Sci U S A. 2013;110(42):17089-17094. doi:10.1073/pnas.1308091110 24082094 PMC3801007

[42] Tamnes CK, Herting MM, Goddings A-L, . Development of the cerebral cortex across adolescence: a multisample study of inter-related longitudinal changes in cortical volume, surface area, and thickness. J Neurosci. 2017;37(12):3402-3412. doi:10.1523/JNEUROSCI.3302-16.2017 28242797 PMC5373125

[43] Miller KL, Alfaro-Almagro F, Bangerter NK, . Multimodal population brain imaging in the UK Biobank prospective epidemiological study. Nat Neurosci. 2016;19(11):1523-1536. doi:10.1038/nn.4393 27643430 PMC5086094

[44] Vidal-Ribas P, Janiri D, Doucet GE, . Multimodal neuroimaging of suicidal thoughts and behaviors in a US population-based sample of school-age children. Am J Psychiatry. 2021;178(4):321-332. doi:10.1176/appi.ajp.2020.20020120 33472387 PMC8016742

[45] Lisdahl KM, Sher KJ, Conway KP, . Adolescent brain cognitive development (ABCD) study: Overview of substance use assessment methods. Dev Cogn Neurosci. 2018;32:80-96. doi:10.1016/j.dcn.2018.02.007 29559216 PMC6375310

[46] Petersen AC, Crockett L, Richards M, Boxer A. A self-report measure of pubertal status: Reliability, validity, and initial norms. J Youth Adolesc. 1988;17(2):117-133. doi:10.1007/BF01537962 24277579

[47] Karoly HC, Callahan T, Schmiege SJ, Ewing SW. Evaluating the Hispanic paradox in the context of adolescent risky sexual behavior: the role of parent monitoring. J Pediatr Psychol. 2016;41(4):429-440. doi:10.1093/jpepsy/jsv039 25972373 PMC4829736

[48] Arthur MW, Briney JS, Hawkins JD, Abbott RD, Brooke-Weiss BL, Catalano RF. Measuring risk and protection in communities using the Communities That Care Youth Survey. Eval Program Plann. 2007;30(2):197-211. doi:10.1016/j.evalprogplan.2007.01.009 17689325

[49] Modabbernia A, Janiri D, Doucet GE, Reichenberg A, Frangou S. Multivariate Patterns of Brain-Behavior-Environment Associations in the Adolescent Brain and Cognitive Development Study. Biol Psychiatry. 2021;89(5):510-520. doi:10.1016/j.biopsych.2020.08.014 33109338 PMC7867576

[50] Heeringa SG, Berglund PA. A guide for population-based analysis of the Adolescent Brain Cognitive Development (ABCD) Study baseline data. bioRxiv. Preprint posted online February 10, 2020. doi:10.1101/2020.02.10.942011

[51] Veale JF. Edinburgh Handedness Inventory - Short Form: a revised version based on confirmatory factor analysis. Laterality. 2014;19(2):164-177. doi:10.1080/1357650X.2013.783045 23659650

[52] Benjamini Y, Hochberg Y. Controlling the false discovery rate: a practical and powerful approach to multiple testing. J R Stat Soc B. 1995;57(1):289-300. doi:10.1111/j.2517-6161.1995.tb02031.x

[53] Abreu-Villaça Y, Seidler FJ, Tate CA, Slotkin TA. Nicotine is a neurotoxin in the adolescent brain: critical periods, patterns of exposure, regional selectivity, and dose thresholds for macromolecular alterations. Brain Res. 2003;979(1-2):114-128. doi:10.1016/S0006-8993(03)02885-3 12850578

[54] Dickinson DK, Golinkoff RM, Hirsh-Pasek K. Speaking out for language: why language is central to reading development. Educ Res. 2010;39(4):305-310. doi:10.3102/0013189X10370204

[55] Friederici AD, Gierhan SM. The language network. Curr Opin Neurobiol. 2013;23(2):250-254. doi:10.1016/j.conb.2012.10.002 23146876

[56] Ivanova MV, Zhong A, Turken A, Baldo JV, Dronkers NF. Functional contributions of the arcuate fasciculus to language processing. Front Hum Neurosci. 2021;15:672665. doi:10.3389/fnhum.2021.672665 34248526 PMC8267805

[57] Turken AU, Dronkers NF. The neural architecture of the language comprehension network: converging evidence from lesion and connectivity analyses. Front Syst Neurosci. 2011;5:1. doi:10.3389/fnsys.2011.00001 21347218 PMC3039157

[58] Brown TT, Kuperman JM, Chung Y, . Neuroanatomical assessment of biological maturity. Curr Biol. 2012;22(18):1693-1698. doi:10.1016/j.cub.2012.07.002 22902750 PMC3461087

[59] Akkermans SEA, van Rooij D, Rommelse N, . Effect of tobacco smoking on frontal cortical thickness development: A longitudinal study in a mixed cohort of ADHD-affected and -unaffected youth. Eur Neuropsychopharmacol. 2017;27(10):1022-1031. doi:10.1016/j.euroneuro.2017.07.007 28764867 PMC5623136

[60] Dai H, Ramos AK, Faseru B, Hill JL, Sussman SY. Racial disparities of e-cigarette use among US youths: 2014-2019. Am J Public Health. 2021;111(11):2050-2058. doi:10.2105/AJPH.2021.306448 34554815 PMC8630507

[61] National Cancer Institute. A socioecological approach to addressing tobacco-related health disparities. National Cancer Institute Tobacco Control Monograph 22. Updated September 24, 2020. Accessed July 20, 2022. https://cancercontrol.cancer.gov/brp/tcrb/monographs/monograph-22

[62] Compton WM, Dowling GJ, Garavan H. Ensuring the Best Use of Data: The Adolescent Brain Cognitive Development Study. JAMA Pediatr. 2019;173(9):809-810. doi:10.1001/jamapediatrics.2019.2081 31305867 PMC8056387

[63] Brener ND, Billy JO, Grady WR. Assessment of factors affecting the validity of self-reported health-risk behavior among adolescents: evidence from the scientific literature. J Adolesc Health. 2003;33(6):436-457. doi:10.1016/S1054-139X(03)00052-1 14642706

