# Supplementary material for: Longitudinal Assessments of Neurocognitive Performance and Brain Structure Associated With Initiation of Tobacco Use in Children, 2016 to 2021
Source: JAMA Netw Open. 2022 Aug 10;5(8):e2225991. doi: 10.1001/jamanetworkopen.2022.25991 (PMC9366547; doi:10.1001/jamanetworkopen.2022.25991)
Supplement: Supplement. — eMethods. Participant Inclusion and Exclusion Criteria eTable 1. Participant Characteristics eTable 2. Comparison of sMRI Morphometric Measures Within Cerebral Hemisphere Between Tobacco Ever Users and Nonusers eTable 3. Region of Interest Analysis of Cortical Volume Between Tobacco Ever Users and Nonusers eTable 4. Sensitivity Analysis of Cognitive Performances and sMRI Morphometric Measures With Adjustment of Intracranial Volume eReferences [file jamanetwopen-e2225991-s001.pdf]

Supplementary Online Content

Dai HD, Doucet GE, Wang Y, et al. Longitudinal assessments of neurocognitive performance and brain structure associated with initiation of tobacco use in children, 2016 to 2021. *JAMA Netw Open*. 2022;5(8):e2225991. doi:10.1001/jamanetworkopen.2022.25991

eMethods. Participant Inclusion and Exclusion Criteria
eTable 1. Participant Characteristics
eTable 2. Comparison of sMRI Morphometric Measures Within Cerebral Hemisphere Between Tobacco Ever Users and Nonusers
eTable 3. Region of Interest Analysis of Cortical Volume Between Tobacco Ever Users and Nonusers
eTable 4. Sensitivity Analysis of Cognitive Performances and sMRI Morphometric Measures With Adjustment of Intracranial Volume
References

This supplementary material has been provided by the authors to give readers additional information about their work.

©2022 Dai HD et al. *JAMA Network Open*.

eMethods. Participant Inclusion and Exclusion Criteria:

As illustrated in Figure 1 in the main text, the ABCD study included 11,876 participants enrolled at Wave 1 (2016-2018) and 10,414 participants at 2-year follow-up (2018-2021) with a retention rate of 87.7%. After excluding participants with missing tobacco ever use status and the NIH Toolbox Cognition Battery measures, Waves 1 and 2 included 11,729 and 10,081 participants, respectively, forming the analytical sample of the cognitive performances. After excluding participants with missing MRI, participants with neuroradiology reports of hydrocephalus and herniation, participants with poor MRI image quality, poor FreeSurfer deconstruction, or traumatic brain injury with loss of consciousness, and those with several medical conditions, such as muscular dystrophy, multiple sclerosis, sickle cell anemia, neoplastic disorders, cerebral palsy, diabetes, epilepsy, lead poisoning, cardiac and kidney disorders, the final analytical sample for MRI neuroimaging outcomes included 10,214 and 6,859 participants for Waves 1 and 2, respectively. The selection procedure was based on the protocols in our previous studies and ABCD study MRI quality control guidelines.[1,2]

ABCD participants with complete tobacco use status and NIH-toolkit measured cognition were excluded from the analysis of neuroimaging outcomes for the criteria outlined below. Wave 1 and Wave 2 bear the same exclusionary criteria, while the difference in the initial census was due to participation attrition over the 2-year follow-up period.

<u>Exclusion of participants from brain structural analysis due to technical neuroimaging issues or medical reasons:</u>

- Missing MRI data (Wave 1 n = 115, Wave 2 n = 2,585)
- Paritcipants with neuroradiology reports of hydrocephalus or herniation or other incidental findings meriting clinical referral.

  ABCD Data Structure = MR Findings (abcd_mrfindings02), element =mrif_score >=3, exclude. (Wave 1 n = 444, Wave 2 n = 357)

- Poor MRI image quality defined as no series passing quality control while ignoring protocol compliance.

  ABCD Data Structure = ABCD MRI QC Raw Part 1 (mriqcrp10301), element =iqc_t1_ok_ser=0, exclude. (Wave 1 n = 6, Wave 2 n = 3)

- Imcomplete or poor freeSurfer deconstruction defined as QC score, motion score, pial overestimation score, white matter underestimation score, inhomogenity score, or artifact score.

  ABCD Data Structure = FreeSurfer QC (fsurfqc01), element =fsqc_qc=0 or fsqc_qu_motion>2 or fsqc_qu_pialover>2 or fsqc_qu_wmunder>2 or fsqc_qu_inhomogeneity>2 or fsqc_qu_artifact>2, exclude. (Wave 1 n = 354, Wave 2 n = 124)

- Traumatic Brain Injury with Loss of Consciousness

©2022 Dai HD et al. *JAMA Network Open*.

ABCD Data Structure = ABCD Sum Scores Traumatic Brain Injury (abcd_tbi01), element =tbi_ss_ntbiloc>0, exclude. (Wave 1 n = 133, Wave 2 n = 90)

- Severe medical conditions including muscular dystrophy, multiple sclerosis, sickle cell anemia, neoplastic disorders, cerebral palsy, diabetes, epilepsy, lead poisoning, cardiac and kidney disorders.

  ABCD Data Structure = Medical History and Health Services Utilization (mx01) , element=max(medhx_2l, medhx_2m, medhx_2p, medhx_2e, medhx_2f, medhx_2g, medhx_2h, medhx_2k, medhx_2o, medhx_2j)=1, exclude. (Wave 1 n = 713, Wave 2 n = 471)

Note: These variables are available under aliases in the current release of ABCD data and may change in the future.

©2022 Dai HD et al. *JAMA Network Open*.

eTable 1. Participant Characteristics

		Wave 1			Wave 2 (2-Year Follow-Up)		
Characteristics	**All**	**Non Use**	**Ever Use**	**P-value[a]**	**Non Use**	**Ever Use**	**P-value[a]**
	**n**	11,613	116		9,995	86	
		8,015,361	98,309		6,844,671	74,255	
Age, mean (SE), year	11,729	9.9 (0.6)	10 (0.6)	.18	12.0 (0.7)	12.0 (0.7)	.47
Sex		**Weighted % (95% CI)**		.04	**Weighted % (95% CI)**		<.001
Male	6116	51.1 (50.2-52)	58.8 (51.4-66.2)		51.2 (50.1-52.4)	62.7 (56.8-68.7)	
Female	5613	48.9 (48-49.8)	41.2 (33.8-48.6)		48.8 (47.6-49.9)	37.3 (31.3-43.2)	
Race/ethnicity				.04			.10
White	6112	52.2 (40.5-63.9)	61.3 (46.6-76)		53.8 (41.9-65.7)	65.5 (49.6-81.4)	
Black	1750	13.3 (8-18.5)	13.9 (4.4-23.3)		12 (7-17.1)	10.3 (1-19.5)	
Hispanics	2381	24 (11.2-36.8)	14.5 (0-29.4)		23.8 (11-36.6)	13.8 (0-29.7)	
Asians	252	3.7 (1.8-5.6)	0.7 (0-2.4)		3.6 (1.7-5.5)	1 (0-3.1)	
Other	1232	6.9 (4.8-9)	9.6 (1.8-17.4)		6.8 (4.5-9.1)	9.5 (0-19)	
Parental education level				<.0001			.005
Less than high school	660	6.9 (4.1-9.7)	5.2 (0.2-10.2)		6.4 (3.5-9.2)	4.5 (0-9.8)	
High school diploma or GED	1164	11.6 (9.4-13.8)	16.7 (6.1-27.2)		10.5 (8.3-12.7)	10.5 (1.2-19.9)	
Some college or associate degree	2979	28.7 (25.1-32.3)	48.2 (36.3-60.1)		28.1 (24.1-32)	47.7 (33-62.5)	
Bachelor degree	3200	25.6 (22.1-29.2)	18.4 (13.3-23.6)		26.7 (23.1-30.4)	20.8 (15-26.5)	
Postgraduate degree	3726	27.2 (22.8-31.6)	11.5 (5.4-17.6)		28.3 (23.3-33.3)	16.4 (7.7-25.1)	
Family income				<.001			<.0001
<$25,000	1609	17 (13-21)	27.2 (16.3-38.1)		15.4 (11.2-19.6)	30.5 (20.2-40.8)	
$25,000-$49,999	1560	18.1 (15-21.2)	24.9 (14.9-35)		18.2 (14.8-21.6)	22.4 (11.4-33.4)	
$50,000-$74,999	1485	15.8 (14-17.5)	22.3 (11.3-33.4)		16.1 (14.3-18)	24.3 (13.5-35.1)	
$75,000-$99,999	1552	12.4 (10.5-14.2)	7.9 (3.4-12.3)		12.9 (11-14.8)	9.6 (3.8-15.5)	

©2022 Dai HD et al. *JAMA Network Open*.

$100,000+	4517	27.3 (21.1-33.5)	8.4 (2.7-14.1)		28.4 (21.9-34.8)	8.9 (2.2-15.6)	
Don't know or refuse to answer	1006	9.4 (7.6-11.3)	9.2 (3.3-15.2)		8.9 (7.2-10.7)	4.3 (0-9.3)	
Family Difficulty				<.0001			<.0001
No	10145	83.9 (81.3-86.6)	69.4 (58.2-80.7)		84.9 (82-87.9)	68.8 (56.7-80.9)	
Yes	1584	16.1 (13.4-18.7)	30.6 (19.3-41.8)		15.1 (12.1-18)	31.2 (19.1-43.3)	
Premature				.09			.11
No	9403	81.2 (75.1-87.4)	73.4 (57.7-89.2)		80.9 (74.5-87.4)	70.6 (50.9-90.4)	
Yes	2183	18.8 (12.6-24.9)	26.6 (10.8-42.3)		19.1 (12.6-25.5)	29.4 (9.6-49.1)	
Other Substance Ever Use				<.001			<.001
No	9065	78.3 (75.1-81.5)	40.1 (32.1-48.1)		78 (74.6-81.5)	44.3 (36.8-51.7)	
Yes	2664	21.7 (18.5-24.9)	59.9 (51.9-67.9)		22 (18.5-25.4)	55.7 (48.3-63.2)	

[a]: Rao-Scott Chi-square tests were performed to compare characteristics between tobacco ever users and non-users, accounting for sampling weights and site clustering.

©2022 Dai HD et al. *JAMA Network Open*.

eTable 2. Comparison of sMRI Morphometric Measures Within Cerebral Hemisphere Between Tobacco Ever Users and Nonusers[a]

Weighted Mean (SE)	Wave 1				Wave 2 (2-Year Follow-Up)			
	Non Use (n=10,117)	Tobacco Ever Use (n=97)	Unadjusted B[b]	Adjusted p-value[c]	Non Use (n=6,806)	Tobacco Ever Use (n=53)	Unadjusted B[b]	Adjusted p-value[c]
**Left Hemisphere**								
Total cortical surface area (mm²)	94292 (469.6)	92476 (1293.2)	-2495.3 (865.4)	.004[c]	94696 (470.5)	93374 (1599.4)	-2624.5 (1206.8)	.03
Mean cortical thickness (mm)	2.7 (0)	2.7 (0)	0 (0)	.43	2.7 (0)	2.7 (0)	0 (0)	.13
Total cortical volume (mm³)	297296 (1207.1)	291198 (3764.5)	-8595.5 (2963.7)	.004[c]	293056 (1325.5)	286962 (5424.1)	-10588.7 (3590.0)	.003[c]
Cerebral white matter volume (mm³)	209138 (886.8)	206307 (3032)	-4185.1 (2350.4)	.08	216347 (886)	215285 (4544.4)	-3952.8 (3388.7)	.24
**Right Hemisphere**								
Total cortical surface area (mm²)	94431 (473.8)	92612 (1280.9)	-2519.5 (880.0)	.004[c]	94815 (470.8)	93458 (1623.2)	-2753.0 (1176.9)	.02
Mean cortical thickness (mm)	2.7 (0)	2.7 (0)	0 (0)	.43	2.7 (0)	2.7 (0)	0 (0)	.13
Total cortical volume (mm³)	297574 (1221.6)	291379 (3937.6)	-8866.6 (2907.5)	.002[c]	293249 (1350.3)	286536 (5623.3)	-11202.1 (3467.2)	.001[c]
Cerebral white matter volume (mm³)	209225 (905.6)	206308 (3103.5)	-4272.2 (2354.9)	.07	216439 (912.8)	215434 (4635.9)	-4163.0 (3287.1)	.20

[a]: Multivariate regression analyses were performed where the dependent variables were sMRI measures in the left and right hemispheres. The independent variable was early age tobacco initiation (ever use of tobacco) measured at Wave 1. The analysis was adjusted by covariates, including age, sex, race/ethnicity, pubertal stage, substance ever use, parental monitoring, school environment, handedness, imaging device manufacturer, and study site. Sampling weights were incorporated to remove the sampling bias.
[b]: Regression coefficients measured the effects of early-age tobacco initiation as changes in sMRI variables between tobacco ever users versus control (no use).
[c]: FDR testing correction was performed to prevent inflation of Type I errors. Adjusted p-value with FDR<0.05 is indicated by this footnote.

©2022 Dai HD et al. *JAMA Network Open*.

eTable 3. Region of Interest Analysis of Cortical Volume Between Tobacco Ever Users and Nonusers[a]

	Cortical Volume (mm^2) Weighted Mean (SE)	Wave 1					Wave 2 (2-Year Follow-Up)				FDR[c]
		Non Use (n=10,117)	Tobacco Ever Use (n=97)	Adjusted B[b]	Adjusted p-value	FDR[c]	Non Use (n=6,806)	Tobacco Ever Use (n=53)	Adjusted B[b]	Adjusted p-value	
Frontal	Superior frontal	57799 (299.1)	56016 (819.7)	-2849.9	<.001	.004	57325 (307.7)	54983 (1219.1)	-3669.3	<.001	.004
	Rostral middle	42501 (219.1)	41773 (719.2)	-1572.3	.01	.03	41793 (241.4)	40822 (966.6)	-1793.2	.02	.04
	Caudal middle	16074 (136.2)	15631 (341.2)	-899.9	.01	.03	16034 (132.5)	15142 (472.7)	-1357.2	.001	.006
	Pars opercularis	11019 (53.1)	10833 (202.2)	-332.7	.19	.23	10939 (55)	10878 (290.4)	-237.2	.47	.51
	Pars triangularis	10810 (37.3)	10740 (213.4)	-139	.47	.48	10615 (49.4)	10564 (248.9)	-207.1	.36	.42
	Pars orbitalis	6955.1 (26.5)	6833.2 (90.2)	-274.2	.02	.04	6837.1 (32)	6644.6 (171.2)	-409.6	.01	.03
	Lateral orbitofrontal	18472 (99.2)	18103 (238.6)	-613.5	.01	.03	18331 (114.5)	17919 (309.1)	-774.3	.009	.02
	Medial orbitofrontal	12664 (93.2)	12410 (170.6)	-511.6	<.001	.004	12492 (97.5)	12156 (212.3)	-628.6	<.001	<.001
	Precentral	31495 (137.3)	30796 (381.2)	-1409.9	<.001	.003	31541 (138)	30849 (617)	-1443.7	<.001	.005
	Paracentral	9353.2 (34.5)	9045 (159.7)	-362.1	.003	.01	9174.7 (36.2)	8840.4 (177.6)	-462.5	.004	.01
	Frontal pole	2987.9 (19.9)	2957.6 (39.7)	-86.6	.11	.16	2903.6 (19.3)	2869.8 (67.6)	-125.2	.04	.07
	Rostral anterior	5520 (33.4)	5465.4 (97.3)	-258.6	.02	.04	5514.3 (32.7)	5415 (144.5)	-304.6	.04	.07
	Caudal anterior	4616 (23.9)	4576.1 (134.8)	-181.9	.12	.16	4617.8 (24.7)	4518.3 (171)	-299.9	.04	.07
Parietal	Superior parietal	34215 (191.5)	33621 (521.9)	-1054.8	.04	.07	33148 (211.2)	32719 (607.3)	-1266.8	.02	.04
	Inferior parietal	35488 (129.7)	34582 (653.2)	-1337.1	.02	.04	34507 (173.3)	32922 (762.1)	-2356.3	<.001	.004

©2022 Dai HD et al. *JAMA Network Open*.

	Supramarginal	27765 (99.7)	26743 (337.6)	-1390.1	.005	.01	27199 (120.3)	26963 (499.3)	-967.9	.14	.21
	Postcentral	23436 (98.4)	23024 (485.4)	-710.2	.10	.14	22914 (103.2)	22165 (715.6)	-1278.9	.008	.02
	Precuneus	25397 (111.6)	24784 (392.6)	-1122.3	.002	.006	24587 (123)	24004 (377.1)	-1334.8	<.001	.004
	Posterior cingulate	8006.1 (28.6)	7869.7 (122)	-227.9	.06	.09	7859.7 (32.8)	7760.5 (138.1)	-259.2	.12	.18
	Isthmus cingulate	6277.5 (25.8)	6194.6 (117.8)	-180	.06	.09	6133.4 (31.1)	6099 (136.5)	-135.9	.23	.32
Temporal	Superior temporal	30408 (126.3)	29188 (392.2)	-1537.6	<.001	.004	30020 (138.5)	29404 (730.7)	-896	.15	.21
	Inferior temporal	26561 (133.6)	26350 (431.8)	-820.2	.05	.08	26428 (144.7)	25952 (760.8)	-1114.4	.05	.08
	Middle temporal	29594 (123.8)	28379 (516.9)	-1854.2	0	.001	29327 (151.6)	28245 (795.9)	-1761.1	.003	.01
	Banks of superior temporal sulcus	5866.6 (32.1)	5561.8 (119.1)	-352.7	<.001	.004	5756.9 (30.9)	5431.1 (141.4)	-493.1	0	<.001
	Fusiform	22129 (85)	21673 (319.3)	-986.3	<.001	.004	21976 (86)	21373 (514.9)	-1116.2	.002	.006
	Transverse temporal	2627.3 (13.1)	2570.6 (46.8)	-83.5	.14	.18	2587.4 (15)	2574.7 (70.9)	-59.5	.48	.51
	Entorhinal	3705.5 (21.4)	3817 (62.1)	76.4	.44	.47	3757.2 (19.9)	3847.2 (90.3)	9.6	.92	.92
	Temporal pole	5447.5 (25)	5546.2 (74.7)	-66.2	.40	.44	5453.1 (20.9)	5527.6 (113)	-100.4	.35	.42
	Parahippocampal	4355.2 (18.5)	4206.8 (57.7)	-218.3	.002	.006	4362 (20.6)	4231.5 (42.1)	-252	.002	.009
Occipital	Lateral occipital	29078 (168.3)	29066 (449.9)	-547.3	.25	.30	28489 (188.3)	28521 (695.6)	-723.2	.28	.345
	Lingual	15973 (86.7)	15871 (311.8)	-378.5	.15	.19	15686 (91.1)	15909 (306)	-248.7	.48	.51
	Cuneus	7885.3 (47.4)	8081.8 (151.9)	60.5	.70	.70	7678.2 (50.6)	7999.5 (179.6)	247.4	.27	.35
	Pericalcarine	5134.5 (34.8)	5210.3 (76.1)	-114.4	.32	.36	5078.3 (43)	5235.9 (127.6)	41.2	.80	.82

©2022 Dai HD et al. *JAMA Network Open*.

	Insula	15251 (80.6)	15056 (161.1)	-368.3	.08	.12	15240 (76.5)	15011 (236.1)	-546	.02	.04

[a]: Multivariate regression analyses were performed where the dependent variables were cortical volumes in 34 regions of interest. The independent variable was early age tobacco initiation (ever use of tobacco) measured at Wave 1. The analysis was adjusted by covariates, including age, sex, race/ethnicity, pubertal stage, substance ever use, parental monitoring, school environment, handedness, imaging device manufacturer, and study site. Sampling weights were incorporated to remove the sampling bias.
[b]: Regression coefficients measured the effects of early-age tobacco initiation as changes in sMRI variables between tobacco ever users versus control (no use).
[c]: FDR correction was performed across 34 regions to prevent inflation of Type I errors.

©2022 Dai HD et al. *JAMA Network Open*.

eTable 4. Sensitivity Analysis of Cognitive Performances and sMRI Morphometric Measures With Adjustment of Intracranial Volume[a]

NIH Toolbox Cognition Battery Weighted Mean (SE)	Wave 1				Wave 2 (2-Year Follow-Up)			
	No Use (n=11613)	Tobacco Ever Use (n=116)	Adjusted B (SE)[a,b]	Adjusted p-value[c]	No Use (n=10,117)	Tobacco Ever Use (n=86)	Adjusted B (SE)[a,b]	Adjusted p-value[c]
Dimensional Charge Card Sort	92.3 (0.3)	91.5 (1)	-0.1 (1.5)	.95	NA	NA	NA	NA
Flanker Inhibitory Control and Attention	93.8 (0.3)	94 (0.7)	0.7 (1.0)	.47	99.9 (0.3)	100 (1.4)	0.5 (2.2)	.81
List Sorting Working Memory	96.1 (0.5)	93.9 (1)	-1.2 (1.3)	.36	NA	NA	NA	NA
Oral Reading Recognition	90.6 (0.2)	88.7 (0.6)	-2.9 (1.5)	.06	94.7 (0.3)	93.1 (0.5)	-1.2 (0.8)	.12
Pattern Comparison Process Speed	88 (0.5)	84.8 (1.2)	-3.5 (1.6)	.04	103.3 (0.5)	101 (1.7)	-0.2 (0.4)	.57
Picture Sequence Memory	102.6 (0.4)	100.1 (0.8)	-2.7 (1.3)	.05	108.5 (0.4)	104.7 (2)	-4.6 (2.1)	.04
Picture Vocabulary Tests	84 (0.5)	82 (0.8)	-2.3 (0.6)	.001[c]	88.5 (0.5)	87 (1.1)	-2.6 (0.8)	.003[c]
Crystalized Cognition Composite Score	86 (0.3)	83.8 (0.5)	-1.7 (0.5)	.004[c]	90.5 (0.4)	89.3 (0.8)	-1.9 (0.9)	.05
Fluid Cognition Composite Score	91.2 (0.5)	88.7 (0.7)	-1.8 (1.3)	.16	NA	NA	NA	NA
Total Cognition Composite Score	85.8 (0.5)	83 (0.6)	-2.2 (1.0)	.04	NA	NA	NA	NA
**Whole Brain Measures**								
Total cortical surface area (mm^2)	188723 (942.5)	185088 (2570.2)	-690.8 (1065.8)	.52	189511 (940.5)	186833 (3217)	-3355.5 (1236.7)	.007[c]
Mean cortical thickness (mm)	2.7 (0)	2.7 (0)	0 (0)	.59	2.7 (0)	2.7 (0)	0 (0)	.18
Total cortical volume (mm^3)	594869 (2423.9)	582577 (7694.5)	-3699.2 (3766.3)	.003[c]	586304 (2669.8)	573498 (11038)	-15487.8 (3680.5)	<.001[c]
Subcortical gray matter volume (mm^3)	59777 (162.9)	59114 (600.6)	-375.3 (351.6)	.29	60533 (207.1)	60441 (776.9)	-43.5 (431.1)	.92
Cerebral white matter volume (mm^3)	418363 (1790.6)	412615 (6132)	-3642.1 (2817.3)	.20	432787 (1797.2)	430719 (9166.7)	-2271.7 (3954.2)	.57

©2022 Dai HD et al. *JAMA Network Open*.

[a]: Multivariate regression analyses were performed where the dependent variables were cognitive performance scores listed in the first column. The independent variable was early age tobacco initiation (ever use of tobacco) measured at Wave 1. The analysis was adjusted by covariates, including age, sex, race/ethnicity, pubertal stage, intracranial volume, substance ever use, parental monitoring, school environment, and study site. Sampling weights and site clustering were incorporated in the survey regression analytical procedures for statistical inference at the population level.
[b]: Regression coefficients measured the effects of early-age tobacco initiation as changes in cognitive performance scores between tobacco ever users versus control (no use).
NA: not available. Cognitive evaluation task was not performed in Wave 2.
[c]: FDR correction was performed to prevent inflation of Type I errors. Adjusted p-value with FDR<0.05 is indicated by this footnote.

References

1. Vidal-Ribas P, Janiri D, Doucet GE, et al. Multimodal neuroimaging of suicidal thoughts and behaviors in a US population-based sample of school-age children. *American Journal of Psychiatry*. 2021;178(4):321-332.
2. Modabbernia A, Janiri D, Doucet GE, Reichenberg A, Frangou S. Multivariate Patterns of Brain-Behavior-Environment Associations in the Adolescent Brain and Cognitive Development Study. *Biol Psychiatry*. Mar 1 2021;89(5):510-520. doi:10.1016/j.biopsych.2020.08.014

©2022 Dai HD et al. *JAMA Network Open*.
